# Diversity of *Trichoderma* species associated with the black rot disease of *Gastrodia elata*, including four new species

**DOI:** 10.3389/fmicb.2024.1420156

**Published:** 2024-07-26

**Authors:** Chuwen Ye, Yanbo You, Wenjie Li, Tingting Jing, Minghe Mo, Min Qiao, Zefen Yu

**Affiliations:** Laboratory for Conservation and Utilization of Bio-resources, Key Laboratory for Microbial Resources of the Ministry of Education, Yunnan University, Kunming, Yunnan, China

**Keywords:** taxonomy, *Trichoderma*, plant disease, *Gastrodia elata*, multi-gene phylogeny

## Abstract

**Introduction:**

*Trichoderma* species establish symbiotic relationships with plants through both parasitic and mutualistic mechanisms. While some *Trichoderma* species act as plant pathogenic fungi, others utilize various strategies to protect and enhance plant growth.

**Methods:**

Phylogenetic positions of new species of *Trichoderma* were determined through multi-gene analysis relying on the internal transcribed spacer (ITS) regions of the ribosomal DNA, the translation elongation factor 1-α (*tef1-*α) gene, and the RNA polymerase II (*rpb2*) gene. Additionally, pathogenicity experiments were conducted, and the aggressiveness of each isolate was evaluated based on the area of the cross-section of the infected site.

**Results:**

In this study, 13 *Trichoderma* species, including 9 known species and 4 new species, namely, *T. delicatum, T. robustum, T. perfasciculatum*, and *T. subulatum* were isolated from the diseased tubers of *Gastrodia elata* in Yunnan, China. Among the known species, *T. hamatum* had the highest frequency. *T. delicatum* belonged to the Koningii clade. *T. robustum* and *T. perfasciculatum* were assigned to the Virens clade. *T. subulatum* emerged as a new member of the Spirale clade. Pathogenicity experiments were conducted on the new species *T. robustum, T. delicatum*, and *T. perfasciculatum*, as well as the known species *T. hamatum, T. atroviride*, and *T. harzianum*. The infective abilities of different *Trichoderma* species on *G. elata* varied, indicating that *Trichoderma* was a pathogenic fungus causing black rot disease in *G. elata*.

**Discussion:**

This study provided the morphological characteristics of new species and discussed the morphological differences with phylogenetically proximate species, laying the foundation for research aimed at preventing and managing diseases that affect *G. elata*.

## 1 Introduction

The genus *Trichoderma*, belonging to the Hypocreales in Ascomycota, is widely distributed in different types of habitats (Migheli et al., 2009). They show significant potential for applications in human societal production. In agriculture, *Trichoderma* species as a soil conditioner can produce abundant secondary metabolites to promote plant growth and enhance plant stress tolerance (Degenkolb et al., 2008; Pruksakorn et al., 2010; Cheng et al., 2012). In industry, the improved strain *Trichoderma reesei* QM6a is used as a cellulase-producing strain (Kubicek et al., 2019). However, some *Trichoderma* spp. are potentially pathogenic fungi that can cause infections in humans. Moreover, several *Trichoderma* species can secrete toxins that decrease the yield of large edible fungi, negatively impact human health and economic development (Kim et al., 2012; Sandoval–Denis et al., 2014).

*Gastrodia elata* BI. contains various active components, including *G. elata* alkaloids and *G. elata* polysaccharides, which enhance immune function, alleviate pain, and exhibit anti-inflammatory and antioxidant properties (Fan et al., 2013; Chen et al., 2016; Liu and Huang, 2017). A special symbiotic relationship exists between *G. elata* and *Armillariella mellea* P. Karst, where *G. elata* relies on *Armillariella mellea* P. Karst for nutrient supply (Zeng et al., 2018). However, certain *Trichoderma* species can parasitize on *A. mellea* in symbiosis with *G. elata*, causing *A. mellea* to die, which consequently affects the growth of *G. elata* (Wu and Ma, 1998). Additionally, *T. viride* Pers. and *T. hamatum* Bainier were reported as the pathogens responsible for causing the black rot disease in *G. elata* (Zeng et al., 2007; Han et al., 2017). Zhang et al. (2020) investigated the diseases of *Gastrodia elata* in Xiaocaoba, Yunnan, and Dafang, Guizhou areas and found that the incidence of diseased *Gastrodia elata* ranged from 6% to 17%, with a yield reduction of 10% to 30%. However, *Trichoderma* also served as a natural defender of *G. elata*. Zhang et al. (2020) isolated *T. harzianum* Rifai from diseased *G. elata* tissues, ensuring the normal growth of *G. elata* by controlling the growth of pathogenic fungi in *G. elata* (Zhang et al., 2020). *T. viride* also exhibited antagonistic effects against corn stalk rot pathogens on *G. elata* (Wu and Ma, 1998). Therefore, the connection between *G. elata* and *Trichoderma* spp. is significantly important and complex.

Research on *Trichoderma* species began more than 200 years ago. With the development of sequencing technology, researchers began to incorporate morphological methods into fungal identification (Lieckfeldt and Seifert, 2000; Kullnig–Gradinger et al., 2002). The internal transcribed spacer (ITS), as the molecular barcode for *Trichoderma*, is the primary sequence used for identification (Lieckfeldt and Seifert, 2000; Chaverri et al., 2001; Kullnig–Gradinger et al., 2002; Samuels et al., 2002). Although the ITS sequence can provide preliminary insights into the classification of *Trichoderma* species, a single gene cannot accurately depict interspecies relationships. New molecular barcodes have gradually been introduced into the classification, such as the translation elongation factor 1-α (*tef1*) and the RNA polymerase II (*rpb2*) gene (Berney et al., 2000; Chaverri et al., 2003; Jaklitsch et al., 2013; Jaklitsch and Voglmayr, 2015). The universality of *tef1* and *rpb2* makes them ideal identification tools (Berney et al., 2000; Chaverri et al., 2003). The multi-gene analysis supports the exploration of the diversity of *Trichoderma* species associated with *G. elata*, which provides a theoretical basis for the prevention of *Gastrodia* diseases.

In this study, 93 strains were isolated from the diseased tubers of *G. elata* collected from Yiliang County, Zhaotong City, Yunnan Province. Among these strains, 85 belonged to 9 known species. Four new *Trichoderma* species belonging to three clades were described and illustrated using multi-gene analysis. Additionally, the pathogenicity of the representative species was tested.

## 2 Materials and methods

### 2.1 Sample collection

Approximately more than 100 diseased tubers of *G. elata* were collected from the planting area in Xiaocaoba, Yiliang County, Zhaotong City, Yunnan Province. Some tubers were collected from a planting pond, while others were obtained from markets. The samples were then placed in sterile self-sealing plastic bags, labeled, and transported to the laboratory. These samples were stored at 4°C.

### 2.2 Isolation of *Trichoderma*

The plant tissues were washed to remove soil and debris, and then they were immersed in 75% ethanol (75 mL of ethanol was diluted with water to obtain a total volume of 100 mL) for 2 min. After rinsing three times with sterile water for 30 s each, the tissues were immersed in 1.5% sodium hypochlorite (1.5 g of sodium hypochlorite was dissolved in 100 mL of water) for 2 min, and then the rinsing step was repeated. Subsequently, the tissues were air-dried. The plant tissues were cut into slices of 5 mm × 5 mm dimensions and were placed on potato dextrose agar (PDA) plates (PDA: 200 g potato, 20 g dextrose, 18 g agar, and 1,000 mL distilled water) at 25°C. After mycelium growth, the strains with morphological characteristics of *Trichoderma*, such as abundant and fluffy hyphae, and yellow-green or green colonies, were selected for transferring into PDA plates for initial purification culture and identification. The pure and dried cultures were deposited in the herbarium of the Laboratory for Conservation and Utilization of Bio-Resources, Yunnan University, Kunming, Yunnan, China (YMF).

### 2.3 Morphology observation

Perforate PDA plates were covered with mycelium using a 5 mm diameter punch. Inoculate circular mycelial plugs were secured onto the PDA medium, cornmeal agar (CMA) plates (CMA: 20 g of cornmeal, 18 g of agar, and 1,000 mL of distilled water), and synthetic nutrient-poor agar (SNA) plates (SNA: 1 g of KH_2_PO4, 1 g of KNO3, 0.5 g of MgSO4, 0.5 g of KCl, 0.2 g of glucose, 0.2 g of sucrose, 18 g of agar, and 1,000 mL of distilled water). The plates were incubated at 25°C, 30°C, and 35°C under alternating 12 h light and 12 h darkness conditions. The colony radius was recorded after 3 days, and the time taken for complete colony coverage was noted. Microscopic observations of hyphae, conidiophores, phialides, conidia, and other microscopic structures were performed using an Olympus BX51 microscope (Tokyo, Japan) connected to a DP controller digital camera. At least 30 datasets were measured for each structure. Colonies were photographed after 7 days of production, and conidia were photographed after 14 days of production.

### 2.4 DNA extraction, Polymerase Chain Reaction (PCR) amplification, and sequencing

The 0.5 g fungal mycelia were collected and transferred into a 1.5 mL microcentrifuge tube with a 0.7–0.8 mL of lysis buffer [7 mol/L Urea, 50 mmol/L Tris-HCl, 62.5 mmol/L NaCl, 10 g/L Sodium Dodecyl Sulfate (SDS)]. The mixture was centrifuged at 12,000 r/min for 5 min, and the aqueous phase was transferred into a new 1.5 mL tube. An equal volume of the DNA extract (phenol/chloroform/isoamyl alcohol, 25:24:1) was added to the homogenates. The mixture was centrifuged at 12,000 r/min for 5 min, and the aqueous phase was transferred into a new 1.5 mL tube. The homogenates containing DNA were re-extracted by adding an equal volume of isopropanol and 1/10 volume of 3 mol/L NaAc. The mixture was placed at −20°C for 20 min and then centrifuged at 12,000 r/min for 5 min. The aqueous phase was then discarded. The DNA pellet was washed twice with 70% ethanol (70 mL of ethanol was diluted with water to obtain a total volume of 100 mL) in order to precipitate the pellet further. Afterward, it was dried and resuspended in 50 μL of H_2_O for PCR analysis (Sun et al., 2000; Liu et al., 2005). Fragments of ITS, *rpb2*, and *tef1* were amplified with three primer pairs: ITS4 and ITS5 for ITS (White et al., 1990), frpb2-5f and frpb2-7cr for rpb2 (Liu et al., 1999), and EF1-728F (Carbone and Kohn, 1999) and TEF1LLErev (Jaklitsch et al., 2005) for tef1. A 25 μL reaction volume contained 1.0 μL of DNA template, 1.0 μL of each forward and reverse primer, 12.5 μL of 2 × MasterMix (Tiangen Biotech), and 9.5 μL of ddH_2_O. The PCR thermal cycle programs are shown in Table 1. PCR products were purified using the PCR product purification kit (Biocolor Bioscience and Technology Co., Shanghai, China), and forward and reverse sequencing was carried out on an ABI 3730 XL DNA sequencer (Applied Biosystems, Foster City, California) with the primers used during the PCR amplification. The sequences were deposited in the GenBank database at the National Center for Biotechnology Information (NCBI). The accession numbers are listed in Table 2.

**Table 1:** PCR primers and conditions.

**Gene/locus**	**Primer**	**Sequence (5^′^-3^′^)**	**PCR conditions**
ITS	ITS5	GGAAGTAAAAGTCGTAACAAGG (White et al., 1990)	5 min at 94°C; 30 cycles of 30s at 95°C, 30s at 55°C, 30s at 72°C, and 5 min at 72°C
	ITS4	TCCTCCGCTTATTGATATGC (White et al., 1990)	
*tef1*	EF1-728F	CATCGAGAAGTTCGAGAAGG (Carbone and Kohn, 1999)	5 min at 94°C; 35 cycles of 45s at 95°C, 45s at 55°C, 1 min at 72°C, and 5 min at 72°C
	TEF1LLErev	AACTTGCAGGCAATGTGG (Jaklitsch et al., 2005)	
*rpb2*	fRPB2-5f	GA(T/C) GA(T/C) (A/C) G(A/T) GATCA(T/C) TT(T/C) GG (Chen and Zhuang, 2017)	5 min at 94°C; 35 cycles of 1 min at 95°C, 1 min at 55°C, 90s at 72°C, and 5 min at 72°C
	fRPB2-7cR	CCCAT(A/G) GCTTG(T/C) TT(A/G) CCCAT (Chen and Zhuang, 2017)	

**Table 2:** Strains and their GenBank accession numbers.

**Species**	**Strain**	**GenBank accession number**		
		**ITS**	***rpb2***	***tef1***
*Protocre apallida*	CBS 299.78	MH861137	EU703948	EU703900
*Protocrea farinosa*	CBS 121551	MH863119	EU703935	EU703889
*T. afroharzianum*	CBS 124620^*^	FJ442265	FJ442691	FJ463301
*T. afroharzianum*	GJS 04-193	FJ442233	FJ442709	FJ463298
*T. asiaticum*	YMF 1.00168	MH262582	MH262575	MH236492
*T. asiaticum*	YMF 1.00352^*^	MH113930	MH158994	MH183183
*T. atroviride*	TRS26	KJ786751	KP009054	KJ786832
*T. atroviride*	CBS 119499	FJ860726	FJ860518	FJ860611
*T. azevedoi*	CEN 1422^*^	MK714902	MK696821	MK696660
*T. azevedoi*	CEN 1423	MK714903	MK696822	MK696661
*T. byssinum*	HMAS 248838^*^	KY687921	KY687977	KY688035
*T. byssinum*	HMAS 248839	KY687922	KY687978	KY688036
*T. caribbaeum*	GJS 97-3^*^	DQ313131	KJ665246	DQ284977
*T. caribbaeum*	GJS 98-43	DQ313139	NA	DQ284976
*T. ceramicum*	CBS 114576^*^	FJ860743	FJ860531	FJ860628
*T. ceramicum*	GJS 88-70	AY737764	AF545510	AF534593
*T. chlamydosporicum*	HMAS 248851	KY687934	KY687990	KY688053
*T. crassum*	TRS113	KP009300	KP009102	KP008865
*T. dacrymycellum*	WU 29044^*^	FJ860749	FJ860533	FJ860633
*T. dorotheae*	GJS 99-202^*^	DQ313144	EU248602	DQ307536
*T. dorothopsis*	HZA5	MH624140	MH647795	MK850827
*T. dorothopsis*	HZA15	MH624150	MH647805	MH647805
*T. hainanense*	HMAS 248837^*^	KY687920	KY687976	KY688033
*T. hainanense*	HMAS 248866	KY687949	KY688004	KY688034
*T. harzianum*	CBS 226.95^*^	AJ222720	AF545549	AF348101
*T. harzianum*	GJS 05-107	FJ442679	FJ442708	FJ463329
*T. hirsutum*	HMAS 248834^*^	KY687916	KY687972	KY688029
*T. hirsutum*	HMAS 248859	KY687942	KY687998	KY688030
*T. hunanense*	HMAS 248841^*^	NR_154571	KY687980	KY688039
*T. hunanense*	HMAS 248867	KY687950	KY688005	KY688040
*T. istrianum*	S123	NA	KJ665280	KJ665521
*T. istrianum*	CBS 130539^*^	NA	KJ665281	KJ665523
*T. koreanum*	SFC20130926-S008	NA	MH025989	MH025983
*T. koreanum*	SFC20131005-S066^*^	MH050352	MH025988	MH025979
*T. lentinulae*	CGMCC 3.19848	MN594470	MN605868	MN605879
*T. lentinulae*	HMAS 248256^*^	MN594469	MN605867	MN605878
*T. linzhiense*	HMAS 248846^*^	KY687929	KY687985	KY688047
*T. linzhiense*	HMAS 248874	KY687957	KY688011	KY688048
*T. longipile*	CBS 120953^*^	NA	FJ860542	FJ860643
*T. longipile*	CBS 135570	NA	KJ665292	KJ665556
*T. longisporum*	HMAS 248843^*^	KY687926	KY687982	KY688043
*T. longisporum*	HMAS 248868	KY687951	KY688006	KY688044
*T. martiale*	CBS 123052^*^	DQ315454	EU248597	DQ307534
*T. neocrassum*	HMAS 275617^*^	NA	MH746772	MH746776
*T. nigricans*	T32450	NA	OP357958	OP357973
*T. nigricans*	T32794	NA	OP357960	OP357975
*T. nigricans*	T32781^*^	NA	OP357959	OP357974
*T. obovatum*	YMF 1.06211^*^	MN977803	MT038432	MT070144
*T. obovatum*	YMF 1.06212	MN977804	MT038433	MT070143
*T. ochroleucum*	Strain	FJ860793	FJ860556	FJ860659
*T. paratroviride*	S385^*^	NA	KJ665321	KJ665627
*T. paratroviride*	S489	NA	KJ665322	KJ665628
*T. paraviride*	YMF 1.04628^*^	MK775514	MK775513	MK775508
*T. peberdyi*	CEN1425	MK714905	MK696824	MK696663
*T. peberdyi*	CEN1426^*^	MK714906	MK696825	MK696664
*T. pinicola*	KACC 48486 ^*^	MH050354	MH025993	MH025981
*T. pinicola*	SFC20130926-S014	NA	MH025991	MH025978
*T. polypori*	HMAS 248855^*^	KY687938	KY687994	KY688058
*T. polypori*	HMAS 248861	KY687944	KY688000	KY688059
*T. rifaii*	CBS 130746^*^	FJ442663	NA	FJ463324
*T. rifaii*	DIS 337F	FJ442621	FJ442720	FJ463321
*T. rugulosum*	SFC20180301-001^*^	MH050353	MH025986	MH025984
*T. rugulosum*	SFC20180301-002	NA	MH025987	MH025985
*T. shennongjianum*	HMAS 245009	KX060605	KT735259	KT735253
*T. silvae-virgineae*	CBS 120922	FJ860836	FJ860587	FJ860696
*T. simile*	YMF 1.06201^*^	MN977793	MT052184	MT070154
*T. simile*	YMF 1.06202	MN977794	MT052185	MT070153
*T. simplex*	HMAS 248842^*^	KY687925	KY687981	KY688041
*T. simplex*	HMAS 248860	KY687943	KY687999	KY688042
*T. spirale*	DIS 173A	FJ442217	FJ442705	FJ463371
*T. spirale*	E425	NA	MK044189	MK044096
*T. stramineum*	CBS 114248^*^	AY737765	AY391945	AY737746
*T. stramineum*	TAMA 0425	AB856609	AB856748	AB856675
*T. subazureum*	YMF 1.06207^*^	MN977799	MT052190	MT070148
*T. subuliforme*	YMF 1.06204^*^	MN977796	MT052187	MT070151
*T. subuliforme*	YMF 1.06205	MN977797	MT052188	MT070150
*T. subuliforme*	YMF 1.06206	MN977798	MT052189	MT070149
*T. texanum*	LESF551	HQ608136	KT278920	KT278988
*T. tibetica*	YMF 1.05583^*^	MK779177	MK779178	MK779179
*T. tomentosum*	DAOM 178713a^*^	DQ085432	AF545557	EU279969
*T. uncinatum*	YMF 1.04622^*^	MK795994	MK795990	MK795986
*T. undatipile*	HMAS 248857^*^	KY687940	KY687996	KY688057
*T. undatipile*	HMAS 248854	KY687937	KY687993	KY688056
*T. variegatum*	YMF 1.07532	OQ517964	OQ559129	OQ559120
*T. variegatum*	YMF 1.07533^*^	OQ517965	NA	OQ559121
*T. variegatum*	YMF 1.07534	OQ517966	OQ559130	OQ559122
*T. vermifimicola*	CGMCC 3.19850	MN594472	MN605870	MN605881
*T. vermifimicola*	HMAS 248255^*^	MN594473	MN605871	MN605882
*T. virens*	HBG1-2	OQ734732	OQ791370	OQ791372
*T. virens*	HNG1-1	OQ734731	OQ791369	OQ791371
*T. virens*	CBS 249.59^*^	AF099005	AF545558	AF534631
*T. viride*	CBS 119325^*^	DQ677655	EU711362	DQ672615
*T. viride*	TRS575	KP009372	KP009081	KP008931
*T. robustum*	YMF 1.07895	NA	PP388987	PP405572
*T. robustum*	YMF 1.08101^*^	PP213136	PP388988	PP405573
*T. delicatum*	YMF 1.07893	PP145820	PP388989	PP405574
*T. delicatum*	YMF 1.08102^*^	PP213137	PP388990	PP405575
*T. perfasciculatum*	YMF 1.07894^*^	PP213138	PP388992	PP405577
*T. perfasciculatum*	YMF 1.08103	PP213139	PP388991	PP405576
*T. subulatum*	YMF 1.07535^*^	OQ517967	OQ559131	OQ559123
*T. subulatum*	YMF 1.08150	PP213140	PP388993	NA

NA, not applicable. ^*^, type strains.

### 2.5 Phylogenetic analyses

Preliminary Basic Local Alignment Search Tool (BLAST) searches using ITS sequences of isolates against NCBI and UNITE databases have identified both the known species and suspected new species. The suspected new species were subjected to a BLAST search from GenBank, and the top three species in terms of similarity were selected as reference strains, preferably including the type strain of each species. The results indicated that the rpb2 similarity failed to meet the ≥99% standard, and the tef1 similarity failed to meet the ≥97% standard (Table 3). The rpb2 and tef1 sequences did not conform to the standards for the known species. This discrepancy indicates that further analysis is required to determine the systematic position of the suspected new species (Cai et al., 2022).

**Table 3:** The similarity of *rpb2* and *tef1* between the query species and the related species.

**Query species**	**Strain**	***rpb2***		***tef1***	
		**Related species**	**Idensity (%)**	**Related species**	**Idensity (%)**
*T. robustum*	YMF 1.07895	*T. koreanum*	99.14	*T. linzhiense*	94.46
		*T. linzhiense*	97.2	*T. polypori*	93.63
		*T. simplex*	95.36	*T. ceramicum*	93.23
	YMF 1.08101^*^	*T. koreanum*	99.57	*T. linzhiense*	94.46
		*T. linzhiense*	97.62	*T. polypori*	93.63
		*T. simplex*	95.57	*T. ceramicum*	93.23
*T. delicatum*	YMF 1.07893	*T. texanum*	96.89	*T. tibetica*	95.82
		*T. dorothopsis*	96.24	*T. ochrolecum*	93.65
		*T. istrianum*	96.13	*T. viride*	93.46
	YMF 1.08102^*^	*T. texanum*	96.98	*T. tibetica*	95.74
		*T. dorothopsis*	96.33	*T. ochrolecum*	93.57
		*T. istrianum*	96.11	*T. viride*	93.29
*T. perfasciculatum*	YMF 1.07894^*^	*T. ceramicum*	94.92	*T. chlamydosporum*	93.8
		*T. linzhiense*	94.82	*T. ceramicum*	93.34
		*T. shennongjianum*	94.6	*T. byssinum*	93.5
	YMF 1.08103	*T. ceramicum*	94.82	*T. chlamydosporum*	92.27
		*T. linzhiense*	94.71	*T. byssinum*	92.37
		*T. shennongjianum*	94.49	*T. ceramicum*	92.14
*T. subulatum*	YMF 1.07535^*^	*T. hunanense*	98.46	*T. hunanense*	95.98
		*T. subuliforme*	96.14	*T. longisporum*	94.21
		*T. longisporum*	95.81	*T. hainanense*	91.26
	YMF 1.08150	*T. hunanense*	98.35	*T. hunanense*	95.73
		*T. subuliforme*	96.14	*T. longisporum*	93.97
		*T. longisporum*	95.7	*T. hainanense*	91.02

Notes: ^*^, type strains.

The research revealed that the new species belonged to three clades: the Spirale clade, the Virens clade, and the Koningii clade. We selected all strains reported in recent years within the Spirale clade as reference strains. For the Virens clade, we selected the main species of this clade as reference strains. In addition, the Helicum and Strictipile clades were selected as secondary references because the identification of some strains was ambiguous. For the Koningii clade, strains with a high sequence similarity were selected as reference strains. Moreover, the main strains of the Atroviride clade were selected as reference strains. *Protocrea farinosa* Berk & Broome (CBS 121551) and *P. pallida* Ellis & Everh (CBS 299.78) were used as outgroups.

Sequence alignment was performed using ClustalX 1.83. Based on the alignment, the sequence was trimmed to the appropriate length using MEGA11. Appending was performed using BioEdit v.7.0. The three genes were merged into a single sequence for subsequent operations. The combined sequence matrix (Fasta file) contained 2,775 characters from each gene (561 from ITS, 932 from *rpb2*, and 1,282 from *tef1*). The alignment data from phylogenetic analyses were deposited in TreeBASE (http://purl.org/phylo/treebase/phylows/study/TB2:S31222).

The systematic position of the new species was determined using Maximum Likelihood (ML) and Bayesian Inference (BI) analyses. The ML analysis was conducted using Iqtree. The command “iqtree -s example.fas -m MF -nt AUTO” was used to select the best model and generate the output. The best model used in this study was TIM2e+R9. Bootstrap values were calculated from 1,000 replicates. After setting the outgroup, Iqtree was run to complete the ML analysis. Bayesian trees were constructed using MrBayes v3.1.2, with the best model chosen through MrModeltest 2.3. The corresponding NEXUS file was prepared, Metropolis-coupled Markov Chain Monte Carlo (MCMC) was initiated for 1,000,000 generations, and every 100 generations were sampled to achieve a final average standard deviation of split frequencies below 0.01. The first 25% of the MCMC samples were discarded, and the remaining data were used to calculate posterior probabilities for Bayesian trees. The trees were visualized in FigTree v1.4, with branches showing Maximum Likelihood Bootstrap Proportions (MLBPs) above 70 and Bayesian Inference Posterior Probabilities (BIPPs) above 0.85.

### 2.6 Pathogenicity

Healthy, fresh *Gastrodia elata* tubers were selected for pathogenicity testing of the strains. Three type strains of the new species (YMF 1.08101, YMF 1.08102, and YMF 1.07894) and two type strains of the known species with a high isolation frequency (*T. hamatum* D5-13-3-2, *T. atroviride* D5-2-2, and *T. harzianum* J2-10-2) were selected. The *Gastrodia elata* tubers were surface-sterilized, according to the method described in Section 2.2, and then dried on a sterile filter paper. Then, each tuber was placed in a Petri dish, measuring 15 cm in diameter and 3 cm in depth, the filter paper sheet was soaked in 10% glycerin at the bottom of the Petri dish to maintain humidity. A U-shaped tube was placed on the filter paper. The healthy *G. elata* tubers were inoculated by placing mycelial plugs (5 mm in diameter), which were cut from the margins of actively growing colonies, into premade holes that were 4 mm in diameter and 2 mm in depth, with the mycelial side facing down on the tubers. Three replicate tubers were inoculated for each isolate, and non-colonized agar plugs were used as negative controls. The Petri dishes were sealed with a plastic wrap, incubated at 25°C, and were observed periodically. After 12 days, pathogens were isolated and identified from each *G. elata* tuber with symptomatic lesions. The aggressiveness of each isolate was evaluated based on the area of the cross-section of the infected site on a continuous scale of 0 to 3, in which 0 indicated no visible lesions, 1 indicated the area of disease lesions up to 1.0 cm^2^, 2 indicated the area of disease lesions from 1.1 to 2.0 cm^2^, and 3 indicated the area of disease lesions >2.0 cm^2^.

## 3 Results

### 3.1 Identification

In this study, 93 strains of *Trichoderma* were isolated and purified from the diseased tubers of *G. elata*. Among these strains, 85 were identified as the known species and 8 were defined as potential new species based on the BLAST search results of the ITS sequence.

Phylogenetic analyses inferred from the ITS sequence were conducted to identify the known species, which included nine species, namely, *T. hamatum, T. atroviride P. Karst, T. harzianum, T. crassum* Bissett, *T. lixii* Chaverri, *T. longipile* Bissett, *T. spirale* Bissett, *T. koningii* Oudem., and *T. scalesiae* Samuels & H.C. Evans (Supplementary Figure 1). The highest isolation frequency was observed in *T. hamatum*, reaching 35.87%. The isolation frequencies of the remaining species were as follows: 18.48% for T. atroviride, 17.39% for *T. harzianum*, 9.78% for *T. crassum*, 5.43% for *T. lixii*, 2.17% for *T. longipile*, and 1.09% for *T. spirale, T. koningii*, and *T. scalesiae* (Supplementary Table 1).

**Figure 1:**
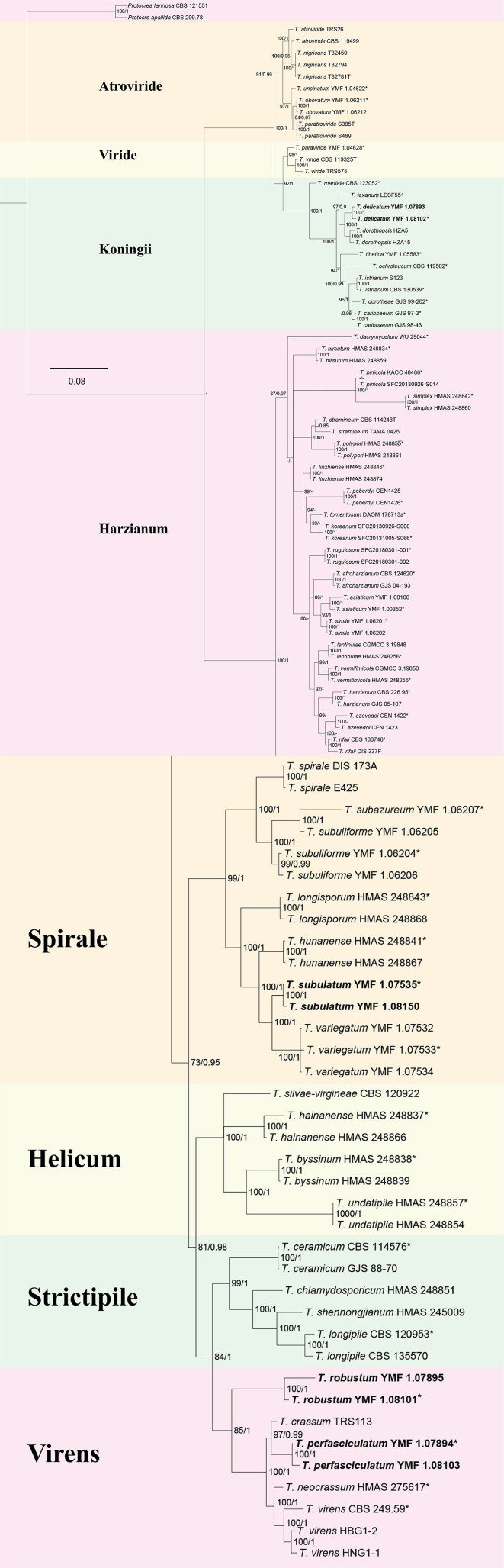
The phylogenetic tree of *Trichoderma* species based on the combined ITS, *tef1*, and *rpb2* gene sequences constructed using the Maximum Likelihood (ML) analysis and Bayesian inference (BI) analysis. The former value near the nodes represents bootstrap support from ML bootstrap over 70%, and the latter value represents Bayesian posterior probabilities over 0.85. *Protocrea farinose* CBS 121551 and *P. pallida* CBS 299.78 were used as outgroups. Bold font indicates newly described species.

The multi-gene analysis involving ITS, *rpb2*, and *tef1* provided evidence for the identification of the new species. The ML analysis demonstrated a similar tree topology, which was consistent with the results obtained from BI analyses (Figure 1). The tree topology exhibited four new species: *T. delicatum* (MLBP/BIPP = 100/1.00), *T. robustum* (MLBP/BIPP = 100/1.00), *T. perfasciculatum* (MLBP/BIPP = 100/1.00), and *T. subulatum* (MLBP/BIPP = 100/1.00). *T. delicatum* belonged to the Koningii clade (MLBP/BIPP = 99/1.00). *T. robustum* and *T. perfasciculatum* belonged to the Virens clade (MLBP/BIPP = 85/1.00). *T. subulatum* was located in the Spirale clade (MLBP/BIPP = 100/1.00). *T. delicatum* was related to *T. dorothopsis* A.A. Tomah and J.Z. Zhang, and *T. texanum* Montoya. *T. robustum* and *T. perfasciculatum* were grouped together and associated with *T. crassum, T. neocrassum* Samuels, and *T. virens* Arx. *T. subulatum* was related to *T. variegatum* Z.F. Yu and T.T. Jing, *T. hunanense* K. Chen and W.Y. Zhuang, and *T. longisporum* K. Chen and W.Y. Zhuang.

### 3.2 Taxonomy

**Figure 2:**
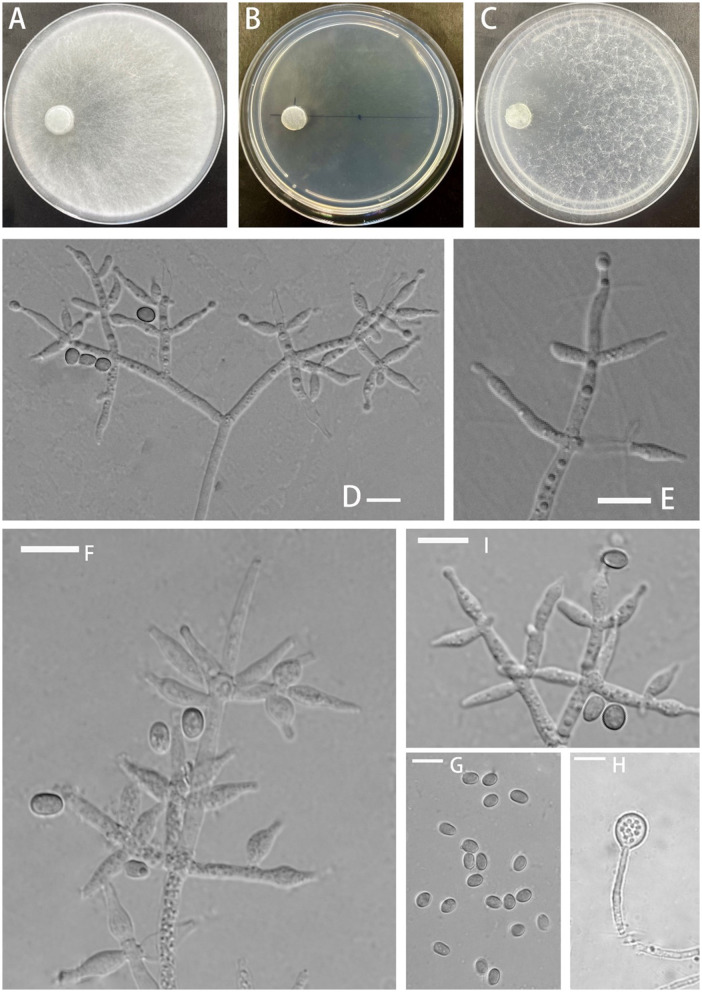
Morphology of *Trichoderma delicatum* (YMF 1.08102). **(A–C)** cultures on PDA plates, 7d; CMA plates, 7d; SNA plates, 7d; 25°C; **(D–F, I)** conidiophores and phialides; **(G)** conidia; and **(H)** chlamydospores. Scale bars: 10 μm **(D–I)**.

***Trichoderma delicatum*** **Z.F. Yu & C.W. Ye, sp. nov**. **Figure 2**

**MycoBank No: 853529**

**Etymology:** Latin, *delicatum*, meaning nice, elegant, fine, slender and referring to conidiophores and conidiogenous cells.

**Description:** The sexual morph of the species is unknown. The asexual morph features conidiophores that are tree-shaped, with a prominent main axis. The branches are irregularly arranged around the main axis, usually including secondary branches. These branches are oriented randomly. Phialide are either ampulliform or narrowly vase-shaped, measuring (−10)10.4–13.0(15.3–) × (−3.4)4.1–4.8(5.4–) μm with a l/w ratio of (−2)2.3–3(3.9–). They are (−2.1)2.4–3.2(3.6–) μm wide at the base and are widest around the middle. Oblong or ellipsoid conidia are green and smooth, measuring (−5.0)5.2–6.0(6.5–) × (−3.3)3.7–4.6(5.4–) μm, with a l/w ratio of (−1.0)1.2–1.5(1.7–). Chlamydospores were observed growing at the tip of hyphae, they are spherical, measuring 8.1–8.9 × 7.3–9.3(−8.6) μm, with a l/w ratio of 1.0–1.2.

**Culture characteristics:** The optimum temperature for growth was 25°C.

Colony radius on the PDA plate after 72 h: 56 mm at 25°C, 53 mm at 30°C, and 25 mm at 35°C, covering the plate after 3.2 days at 25°C. There was a similar growth rate on the PDA plate at 25°C and 30°C. The colony slightly stratified and radial, with abundant aerial hyphae. Thea peripheral hyphae are more numerous than those in center, creating a plush, white appearance. The conidiation time is long. No distinct odor noted.

Colony radius on the CMA plate after 72 h: 26 mm at 25°C, 25 mm at 30°C, and 9 mm at 35°C, covering the plate after 7 days at 25°C. Colony hyaline, aerial hyphae lacking. Conidiation formed after 5.5 days. A little blister-like structures randomly distributed. No distinct odor noted.

Colony radius on the SNA plate after 72 h: 40 mm at 25°C, 29 mm at 30°C, and 20 mm at 35°C, covering the plate after 5.5 days at 25°C. Colony transparent, no zonate, aerial hyphae sparse, abundant at margin, arachnoid. Conidiation started after 5 days, the white mycelium gradually turned green. No obvious odor noted.

**Materials examined:** China, Yunnan Province, Zhaotong City, Yiliang County, Xiaocaoba Town, on diseased *Gastrodia elata*, 25 Oct 2021, T.T. Jing (holotype YMF 1.08102). lbid. (cultures: YMF 1.07893).

**Notes:** Phylogenetically, *T. delicatum* is associated with *T. dorothopsis* and *T. texanum*. *T. delicatum* can be distinguished based on the size of the spores. The spores of *T. dorothopsis* and *T. texanum* are spherical or nearly spherical, with dimensions ranging between 3 and 4 μm (*T. dorothopsis*: (−3.2)3.3–3.9(4.2–) × (−2.9)3.1–3.5(3.6–) μm, *T. texanum*: 1–4 × 1.8–3.1 μm) (Quimi et al., 2016; Ali et al., 2020). In contrast, the spores of *T. delicatum* are ellipsoidal, with a l/w ratio of 1.2–1.5. *T. delicatum* exhibits the fastest growth rate at 25°C, with growth restricted at 30°C and 35°C. *T. dorothopsis* maintains an optimal growth rate at 27–30°C (Ali et al., 2020). The preferred growth temperature for *T. texanum* on the PDA plate is 25°C, while on CMA and the SNA plate, it is 30°C (Quimi et al., 2016). Sporulation of *T. delicatum* is challenging to observe on the PDA plate, and it takes approximately 5.5 days to observe sporulation on the CMA plate. In comparison, *T. dorothopsis* shows sporulation after 4 days on the PDA plate (Ali et al., 2020). Additionally, *T. texanum* exhibits pigment production on the PDA plate, but the phenomenon is not observed in *T. delicatum* and *T. dorothopsis* (Quimi et al., 2016; Ali et al., 2020). Chlamydospores are observable in *T. delicatum* on the PDA plate, and this structure is slightly larger than that of *T. texanum* (Quimi et al., 2016).

**Figure 3:**
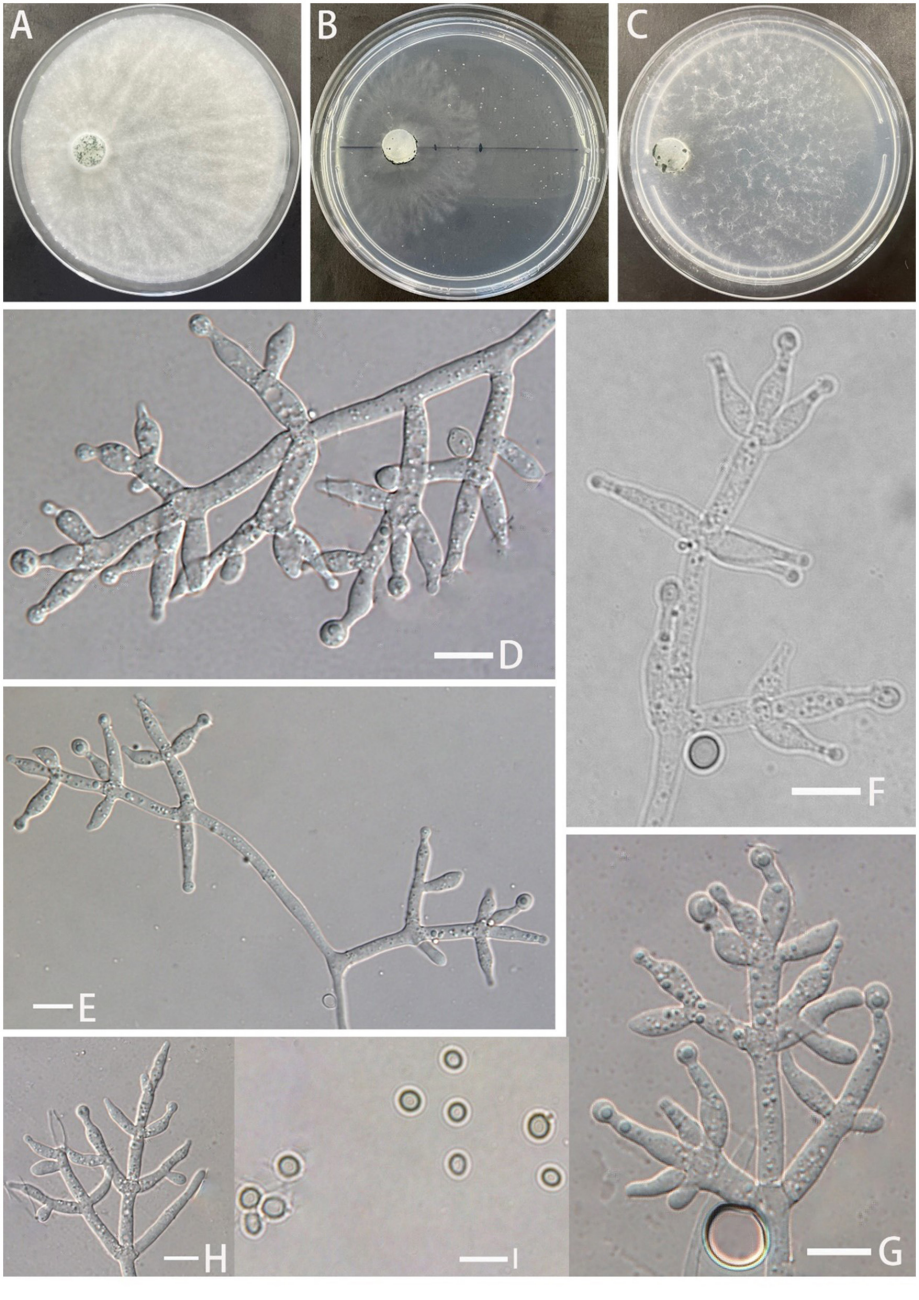
Morphology of *Trichoderma robustum* (YMF 1.08101). **(A–C)** cultures on PDA plates, 7d; CMA plates, 7d; SNA plates, 7d; **(D–H)** conidiophores and phialides; and **(I)** conidia. Scale bars: 10 μm **(D–I)**.

***Trichoderma robustum*** **Z.F. Yu & C.W. Ye, sp. nov**. **Figure 3**

**MycoBank No: 853530**

**Etymology:** Latin, *robustum*, referring to having or exhibiting strength or vigorous conidiophores and conidiogenous cells.

**Description: Sexual morph:** Unknown. **Asexual morph:** Conidiophores with a main axis, mostly secondary branches, tertiary branches also observed, branches asymmetrically distributed on both sides of the main axis toward the tip. Pedicels ampulliform or flask–shaped, 2–3 whorls arising from the ends of the branches, (−8.7)10.1–13.7(15.2–) × (2.5–)2.9–3.5(−3.8) μm, l/w ratio (−2.5)3.0–4.2(5.2–), (−1.9)2.1–2.6(2.8–) μm wide at the base, widest around the middle. Conidia ovate or suborbicular, green, smooth (−4.1)4.3–4.6(4.8–) × (−3.7)4.0–4.3(4.5–) μm, l/w ratio 1.0–1.1(1.3–). No chlamydospores were observed.

**Culture characteristics:** Optimum temperature for growth 25°C.

Colony radius on the PDA plate after 72 h: 36 mm at 25°C, 12 mm at 30°C, and 9 mm at 35°C, covering the plate after 3.8 days at 25°C. Colony slightly stratified, radial, aerial hyphae abundant, plush, and white. The colony producing a green dotted conidiation area from the center outward. No distinct odor noted.

Colony radius on the CMA plate after 72 h: 16 mm at 25°C, 9 mm at 30°C, and 7 mm at 35°C. Colony translucent, aerial hyphae nearly lacking, slow growth, fuzzy stratification. Conidiation starting after 6 days. No distinct odor noted.

Colony radius on the SNA plate after 72 h: 10 mm at 25°C, 8 mm at 30°C, and 5 mm at 35°C, covering the plate after 3.8 days at 25°C. Colony translucent, no ring band, aerial hyphae sparse, flocculent. Conidiation starting after 4 days, green blister-like dots scattered around the plate. No distinct odor noted.

**Materials examined:** China, Yunnan Province, Zhaotong City, Yiliang County, Xiaocaoba Town, on diseased *Gastrodia elata*, 25 Oct 2021, T.T. Jing (holotype YMF 1.08101). lbid. (cultures: YMF 1.07895).

**Notes:** *T. robustum* is phylogenetically associated with *T. crassum, T. virens*, and *T. neocrassum*. Morphologically, *T. robustum* exhibits a prominent main axis with asymmetrical branches distributed on both sides of the main axis, but the branches display uniform intervals. Two to three whorls of phialides arise at the end of each branch, resembling *T. crassum* (2–4 whorls) (Chaverri et al., 2003). The sizes of the phialides in *T. robustum* are comparable to those of *T. crassum* (13.5–15.7 × 4.3–4.6 μm) and are larger than those of *T. virens* (7.9–9.9 × 3.8–4.5 μm) and *T. neocrassum* (7–22 × 2.5–4 μm). Sporulation of *T. robustum* is not observed on PDA plates. In comparison to T. crassum, T. *virens*, and *T. neocrassum, T. robustum* exhibits a significantly slower growth rate (Chaverri et al., 2001, 2003; Zhang and Zhuang, 2019). *T. robustum* is similar to *T. crissum*, which cannot grow at 35°C. However, *T. robustum* shows faster growth on PDA plates than on SNA plates, while the opposite is observed for *T. crassum* (Chaverri et al., 2003). *T. robustum* cannot produce pigments, which distinguishes it from *T. crassum, T. virens*, and *T. neocrassum*. The spores of *T. robustum*, unlike those of *T. crassum* and *T. neocrassum*, are round or nearly round, resembling those of *T. virens* (Chaverri et al., 2001, 2003; Zhang and Zhuang, 2019).

**Figure 4:**
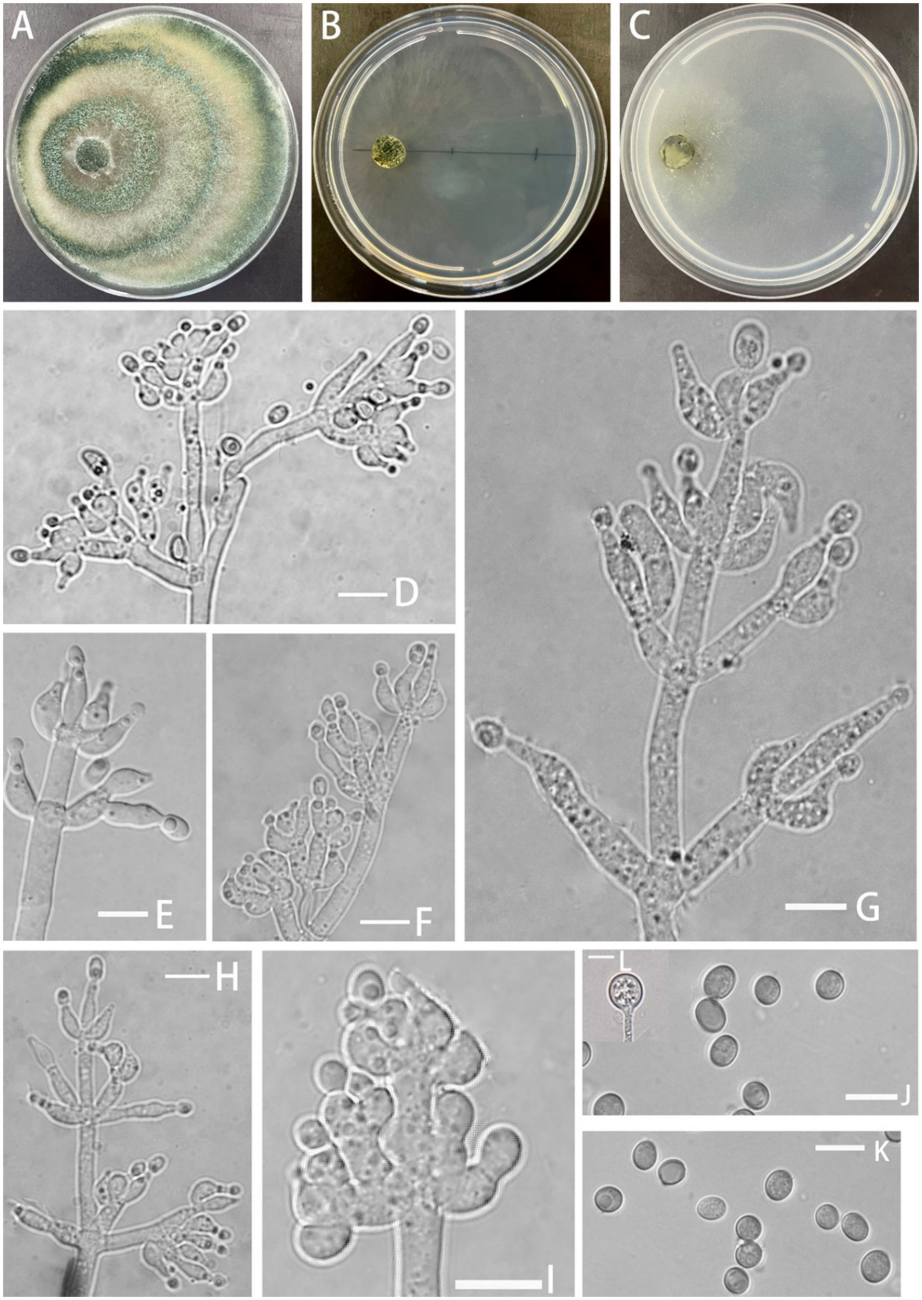
Morphology of *Trichoderma perfasciculatum* (YMF 1.07894). **(A–C)** cultures on PDA plates, 7d; CMA plates, 7d; SNA plates, 7d; **(D–I)** conidiophores and phialides; **(J, K)** conidia; and **(L)** chlamydospores. Scale bars: 10 μm **(D–L)**.

***Trichoderma perfasciculatum*** **Z.F. Yu & C.W. Ye, sp. nov**. **Figure 4**

**MycoBank No: 853531**

**Etymology:** Latin, *perfasciculatum, per-* meaning very, completely + *-fasciculatum* referring to the conidiogenous cells growing and arranging in the fascicles.

**Description: Sexual morph:** unknown. **Asexual morph:** Conidiophores penicillium-like, mostly asymmetrically arranged, main axis conspicuous, with secondary branches, oriented toward the main axis, 2–4 bottlenecks in whorls, clustered. Pedicels ampulla-shaped or narrow ampulla-shaped, partly curved (−10)10.4–13.0(15.3–) × (−3.4)4.1–4.8(5.4–) μm, l/w ratio (−2)2.3–3(3.9–), (−2.1)2.4–3.2(3.6–) μm wide at the base, widest at middle. Conidia ellipsoid, green, and smooth (−5.0)5.2–6.0(6.5–) × (−3.3)3.7–4.6(5.4–) μm, l/w ratio 1.2–1.5. Chlamydospore noted, rounded, terminal, 8.1–8.9 × 7.3–9.3 μm, l/w ratio 1.0–1.2.

**Culture characteristics:** Optimum temperature for growth 25°C.

Colony radius on the PDA plate after 72 h: 54 mm at 25°C, 39 mm at 30°C, and 12 mm at 35°C, covering the plate after 3.8 days at 25°C. Colony dense, white, turning green after conidiation on the 3^rd^ day, producing spores at a fast rate, with obvious circular annular bands, aerial hyphae abundant. No obvious odor noted. Yellow pigment noted.

Colony radius on the CMA plate after 72 h: 25 mm at 25°C, 11 mm at 30°C, and 11 mm at 35°C, covering the plate after 8 days. Colonies were hyaline, radial, mycelium was lacking, slow growing, and stratification not obvious. No distinct odor noted, no chlamydospore observed.

Colony radius on the SNA plate after 72 h: 34 mm at 25°C, 24 mm at 30°C, and 14 mm at 35°C, covering the plate after 4 days at 25°C. Colonies translucent, radial, no ring band, aerial hyphae loose, conidiation starting after 6 days. No obvious odor noted.

**Materials examined:** China, Yunnan Province, Zhaotong City, Yiliang County, Xiaocaoba Town, on diseased *Gastrodia elata*, 25 Oct 2021, T.T. Jing (holotype YMF 1.07895). lbid. (cultures: YMF 1.08103).

**Notes:** From a systematic perspective, *T. perfasciculatum* is closely related to *T. crassum* and associated with *T. virens* and *T. neocrassum*. The growth rate of *T. perfasciculatum* on PDA plates at 25°C is significantly higher than that of *T. crassum* (35–45 μm), *T. virens* (approximately 28μm), and *T. neocrassum* (32–34 μm) within the same clade (Chaverri et al., 2001, 2003; Zhang and Zhuang, 2019). *T. perfasciculatum* and *T. crassum* cannot grow at 35°C, while *T. virens* exhibits the opposite behavior. However, the growth rate of *T. perfasciculatum* on SNA plates is lower than that on PDA plates, and *T. crassum* and *T. virens* present stronger adaptation to SNA plates (Chaverri et al., 2001, 2003). Circular annular bands can be observed in *T. perfasciculatum* and *T. neocrassum* (Zhang and Zhuang, 2019). All four species, *T. perfasciculatum, T. crassum, T. virens*, and *T. neocrassum*, can produce yellow pigments. The phialides of *T. perfasciculatum* and *T. crassum* are more irregular than those of *T. virens*, but all exhibit 2–4 whorls arising from the apex. The phialides of *T. crassum* (l/w radio 3–3.6) are narrower than those of *T. perfasciculatum*. *T. perfasciculatum, T. crassum* (3.7–5.3 × 2.6–3.7 μm, l/w radio 1.4), and *T. neocrassum* (4.5–8 × 3.5–5(7–) μm, l/w radio 1.1–1.6) produce ellipsoidal spores, while the spores of *T. virens* (4.2–4.9 × 3.8–4.2 μm) are smaller and more rounded (Chaverri et al., 2001, 2003; Zhang and Zhuang, 2019).

**Figure 5:**
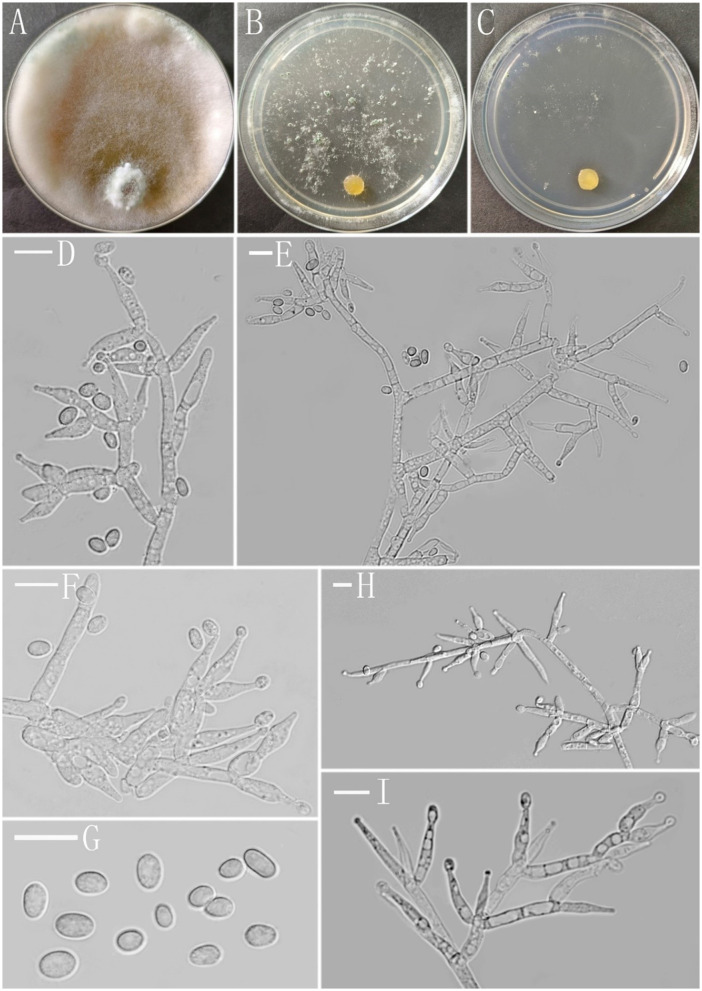
Morphology of *Trichoderma subulatum* (YMF 1.07835). **(A–C)** cultures on PDA plates, 7d; SNA plates, 7d; CMA plates, 7d; **(D–F, H, I)** conidiophores and phialides; and **(G)** conidia. Scale bars: 10 μm **(D–I)**.

***Trichoderma subulatum*** **Z.F. Yu & T.T. Jing, sp. nov**. **Figure 5**

**MycoBank No: 853532**

**Etymology:** Latin, *subulatum*, referring to subulate phialides.

**Description: Sexual morph:** Unknown**. Asexual morph:** Conidiophores asymmetry, often with a main axis, mostly irregularly branched, the terminus bearing phialides, branches often tended toward the conidiophore terminus, rebranching one to three times, the distance between branches relatively large, each branch terminating in a whorl of 2–3 phialides. Phialides subulate often asymmetric, straight or slightly curved, constricted below the tip forming a narrow neck, solitary or in whorls of 2–3(−4), (−7.4)9.4–15.7(16.4–) × (−3.0)3.4–5.1(5.9–) μm, l/w ratio (−1.7)2.1–4.4(4.9–), 2.0–3.3(−3.6) μm wide at the base, widest around the middle. Conidia ellipsoidal to oblong, less globose, green, smooth, (−3.6)4.0–6.1(6.4–) × (−2.7)2.9–3.4(3.6–) μm, l/w ratio (−1.2)1.5–1.9(2.1–).

**Culture characteristics:** Optimum temperature for growth 25°C. Very fast growing colony.

Colony radius on PDA plates after 72 h: 46–47 mm at 25°C, 40–42 mm at 30°C, and 16 mm after 9 days at 35°C, covering the plate after 4 days at 25°C. Colony dense, zonate indistinctly, aerial hyphae abundant, cotton-like, more abundant near the original inoculum and around the periphery of the colony. Conidiation starting after 6 days, formed numerous on aerial hyphae, green. Chlamydospores common, subglobose to globose, smooth, terminal and intercalary, 5.7–10.4 × 5.7–9.2 μm. A distinct odor noted, and yellow pigment noted.

Colony radius on the CMA plate after 72 h: 27–30 mm at 25°C, 25–27 mm at 30°C, and 5 mm after 9 days at 35°C, covering the plate after 6 days at 25°C. Colony homogenous, hyaline, radial, not zonate, aerial hyphae sparse. Conidiation starting after 7 days in pustules, not common, appearing at the colony margin, hemispherical, first white, turning grayish green after 8 days. No chlamydospores observed. A distinct odor noted, and light yellow pigment noted.

Colony radius on the SNA plate after 72 h: 33–35 mm at 25°C, 30–32 mm at 30°C, and 12 mm after 9 days at 35°C, covering the plate at 25°C after 7 days. Colony hyaline, indistinctly zonate, mycelium loose, aerial hyphae floccus. Conidiation formed in pustules after 4 days, pustules appearing around the periphery of the colony, hemispherical, first white, turning deep green after 5 days. No chlamydospores observed. A distinct odor noted, light yellow pigment noted.

**Materials examined:** China, Yunnan Province, Zhaotong City, Yiliang County, Xiaocaoba Town, on diseased *Gastrodia elata*, 25 Oct 2021, T.T. Jing (holotype YMF 1.07535). lbid. (cultures: YMF 1.08150).

**Notes:** Phylogenetically, *T. subulatum* is closely related to *T. hunanense* and *T. variegatum* in the Spirale clade. Compared with the new species, *T. hunanense* produces narrower phialides (3.1–3.9 μm) and shorter and wider conidia [(−3.6)4.2–5.6(7.5–) × 3.1–3.9 μm], forming distinct zonate of pustules on SNA plates (Chen and Zhuang, 2017). *T. variegatum* differs in its longer and wider conidia [(−4.3)4.7–7.4(8.6–) × (−2.7)3.0–4.1(4.3–) μm] and exhibits a lower growth rate on CMA plates (20–22 mm), PDA plates (30–32 mm), and SNA plates (18–22 mm) (Ye et al., 2023). *T. subulatum, T. hunanense*, and *T. variegatum* produce yellow pigment in PDA plates; however, yellow pigment can also be observed in CMA and SNA plates from *T. variegatum*. *T. subulatum* produces a distinct odor in culture, which is not noted in *T. hunanense* and *T. variegatum* (Chen and Zhuang, 2017; Ye et al., 2023).

### 3.3 Pathogenicity

**Figure 6:**
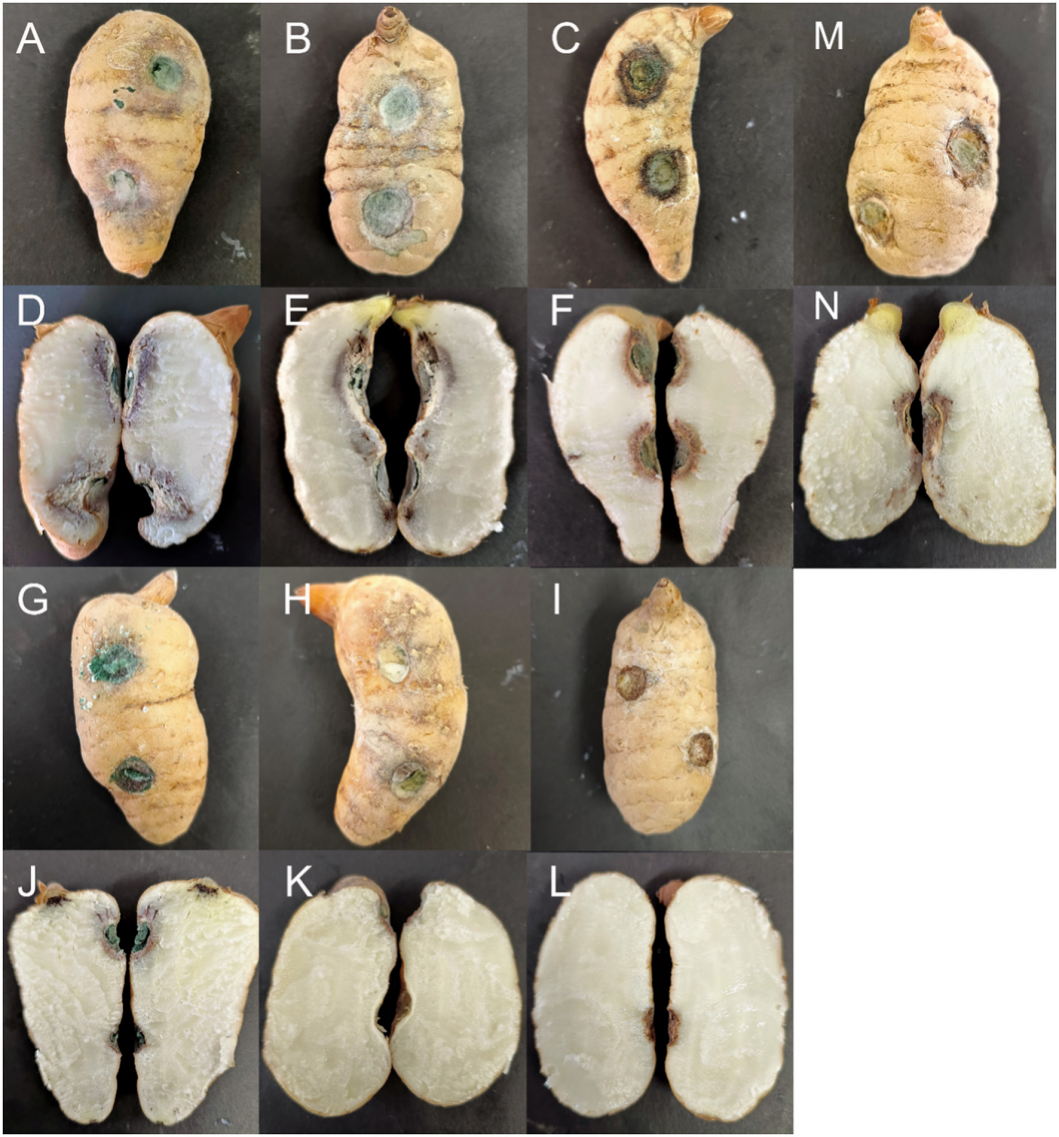
Symptoms of *Gastrodia elata* after its inoculation with testing strains. **(A, D)** *Trichoderma robustum* YMF 1.08101; **(B, E)** *Trichoderma perfasciculatum* YMF 1.07894; **(C, F)** *Trichoderma hamatum* D5-13-3-2; **(G, J)** *Trichoderma atroviride* D5-2-2; **(H, K)** *Trichoderma delicatum* YMF 1.08102; **(I, L)** Control; and **(M, N)** *Trichoderma harzianum* J2-10-2.

After 12 days, the inoculated tubers exhibited varying degrees of infection, while all uninoculated controls remained healthy (Figure 6). The black rot lesions were confined to the area around the inoculation site and did not spread to the area around the uninoculated site. The cross-sections of the inoculation sites on the *G. elata* tubers also demonstrated infection. Dark brown and dry rot areas formed on the superficial skin of the *G. elata* tubers near the inoculation points. The strains that were reisolated from symptomatic tissues were morphologically and phylogenetically identical to the test strains.

The results indicated that the infection abilities of different *Trichoderma* species varied. *T. robustum* (YMF 1.08101) and *T. perfasciculatum* (YMF 1.07894) caused the most severe symptoms, spreading from the inoculation site into the interior of the *Gastrodia elata*. The border between the diseased and healthy tissues appeared purple, and the completely diseased areas near the cortex appeared white and tofu-like. The aggressiveness of these two species reached a scale of 3. *T. hamatum* (D5-13-3-2) appeared moderately pathogenic. The epidermis around the inoculation site darkened, and the mycelium invaded the cortex to form brown spots. The aggressiveness reached a scale of 2. *T. atroviride* (D5-2-2) and *T. harzianum* (J2-10-2) demonstrated weak infection ability, with lesions spreading only to the superficial layers. The aggressiveness reached a scale of 1. *G. elata* inoculated with *T. delicatum* (YMF 1.08102) exhibited no obvious symptoms either on the epidermis or internally. The aggressiveness reached a scale of 0 (Table 4).

**Table 4:** Pathogenicity test results of *Trichoderma* strains isolated from *Gastrodia elata* affected by the black rot disease.

**Isolation/Strain**	**Species**	**Area(cm^2^)**	**Scale**
YMF 1.08101	*Trichoderma robustum*	2.71	3
YMF 1.08102	*Trichoderma delicatum*	0.00	0
YMF 1.07894	*Trichoderma perfasciculatum*	2.81	3
D5-13-3-2	*Trichoderma hamatum*	1.14	2
D5-2-2	*Trichoderma atroviride*	0.47	1
J2-10-2	*Trichoderma harzianum*	0.70	1

## 4 Discussion

Since Wu and Ma (1998) reported that *Trichoderma* could affect the growth of *Gastrodia elata* by parasitizing *Armillariella mellea, T. viride*, and *T. hamatum* were successively reported as pathogenic factors of *G. elata* (Zeng et al., 2007; Han et al., 2017; Zhang et al., 2020). Previously, we also reported that *T. albidum* and *T. variegatum* were isolated from the rot tubers of *G. elata* (Ye et al., 2023). In this study, we reported that 13 species of *Trichoderma*, including *T. atorviride, T. crissum*, and others, were connected with the black rot disease of *G. elata*. *Trichoderma* demonstrated the highest separation frequency of 36.61%. However, it was not detected in an analysis of the association between the brown rot tuber of *G. elata* and soil microbial communities by high—throughput sequencing, in which *Ilyonectria P. Chaverri* & Salgado was the first dominant genus. The genus *Ilyonectria* ranked third with a frequency of isolation of 17.72% (Tang et al., 2020), which may be attributed to variations in methods or regions.

The four new species belonged to the Koningii clade, the Virens clade, and the Spirale clade. The Koningii clade was first proposed as an “aggregate” and was later proposed by researchers as a subclade in the Trichoderma sect. (Rifai, 1969; Bissett, 1991). Lieckfeldt et al. (1998) introduced the *T. koningii* aggregate species on SNA plates, which were characterized by long, narrow spores and slow growth rates. Samuels observed that species morphologically similar to *T. koningii* clustered on a branch in the phylogenetic tree, distinct from *T. viride* and *T. atroviride* (Samuels et al., 2006). Jaklitsch and Voglmayr (2015) named the Viride clade, corresponding to the previously defined *Trichoderma* sect. Qin and Zhuang (2016) reported seven new *Trichoderma* species, and the authors assigned them to different subclades under the Viride clade: Hamatum, Koningii, Neorufum, Rogersonii, Viride, and Viridescent. Zheng et al. (2021) identified two new species belonging to the Koningii clade. The spores of *T. delicatum* were narrower than those of *T. dorothopsis* and *T. texanum*. *T. delicatum* exhibited a slower growth rate on SNA plates. *T. texanum* exhibited pigment production on PDA plates, but the phenomenon was not observed in *T. delicatum* and *T. dorothopsis*.

The Virens clade and the Harzianum clade were previously demonstrated to be closely related. However, researchers now classify the Harzianum/Virens clade as two independent clades (Saravanakumar et al., 2016; Przylucka et al., 2017; Carmen et al., 2021). The representative species in the Virens clade is *T. virens*. Subsequent studies placed *T. crassum* and *T. neocrassum* into the Virens clade, expanding the diversity (Chaverri et al., 2003; Zhang and Zhuang, 2019). Later, Zheng et al. (2021) added *T. inaequilaterale* Z.F. Yu & Y.F. Lv to the Virens clade. Species in this clade generally exhibit faster growth rates and produce gliocladium-like conidiophores and green, smooth spores (Chaverri et al., 2003). The phialides of *T. robustum* and *T. perfasciculatum* are consistent with those of *T. crassum* and *T. virens*, displaying 2–3(−4) whorls arising at the end of the conidiophores (Chaverri et al., 2003). *T. perfasciculatum* exhibits the fastest growth rate in the Virens clade. Neither *T. robustum* nor *T. perfasciculatum* can grow at 35°C, which is consistent with *T. crassum*.

The Spirale clade was initially proposed by Chen and Zhuang (2017). Previous studies showed that *T. spirale* was a distinct terminal branch, but identification using only *tef1* differed from that using *rpb2* and *tef1*. Chaverri et al. (2003) found that T. *spirale* belonged to the Strictipile clade, while Jaklitsch (2009) found that it belonged to the Polysporum clade. Chen and Zhuang (2017) utilized multi-gene analysis composed of ITS, *tef1*, and *rpb2* to discover that *T. hunanense, T. longisporum*, and *T. spirale* were closely related under statistical support. Zheng et al. (2021) later added *T. subuliforme* Z.F. Yu and Y.F. Lv, as well as *T. subazureum* Z.F. Yu and Y.F. Lv, into the Spirale clade. Recently, *T. variegatum* was discovered and grouped into the Spirale clade. Members of the Spirale clade generally form hairy pustules, produce yellow pigments in culture, and have more or less oblong conidia (Chen and Zhuang, 2017), which are typical characteristics of *T. subulatum*. This is the second time that *Trichoderma* in the Spirale clade has been isolated from the rot tuber of *Gastrodia elata*.

The fungal diseases affecting *G. elata* were diverse. Besides *Trichoderma* spp., other pathogens include *Fusarium oxysporum, Poliota praecox* and *Botrytis cinerea* (Zeng et al., 2007), and *Ilyonectria* spp. (Zhang et al., 2020; Qiao et al., 2024). All these studies only reported on pathogens from diseased *G. elata* and not on the interaction between pathogens and *G. elata*. This may be due to the limited distribution of *G. elata*. The cultivation and production of *G. elata* are mainly concentrated in Asian countries, such as China and Korea. In China, Yunnan and Guizhou are the main production areas. Pathogenicity tests revealed differences in the pathogenic capabilities of different *Trichoderma* species. *T. robustum, T. perfasciculatum, T. hamatum, T. atroviride*, and *T. harzianum* showed pathogenic effects on *G. elata*, indicating that *Trichoderma* is indeed a pathogen that causes black rot disease in *G. elata*. However, further research is required to understand how *Trichoderma* causes disease in *G. elata* and whether changes in the fungal community structure of the rhizosphere soil can affect the normal growth of *G. elata*. This study proposed a connection between *Gastrodia elata, Trichoderma*, and black rot disease. It highlighted the impact of *Trichoderma* on *Gastrodia elata* and provided a theoretical basis for future research.

## Data availability statement

The datasets presented in this study can be found in online repositories. The names of the repository/repositories and accession number(s) can be found in the article/Supplementary material.

## Author contributions

CY: Methodology, Formal analysis, Validation, Writing – review & editing, Writing – original draft. YY: Investigation, Writing – original draft. WL: Investigation, Writing – original draft. TJ: Investigation, Writing – original draft. MM: Conceptualization, Funding acquisition, Writing – review & editing. MQ: Conceptualization, Writing – review & editing. ZY: Conceptualization, Resources, Writing – review & editing
