# Supplementary figures and images for: Diversity of Trichoderma species associated with the black rot disease of Gastrodia elata, including four new species
Source: Front Microbiol. 2024 Jul 26;15:1420156. doi: 10.3389/fmicb.2024.1420156 (PMC11310069; doi:10.3389/fmicb.2024.1420156)

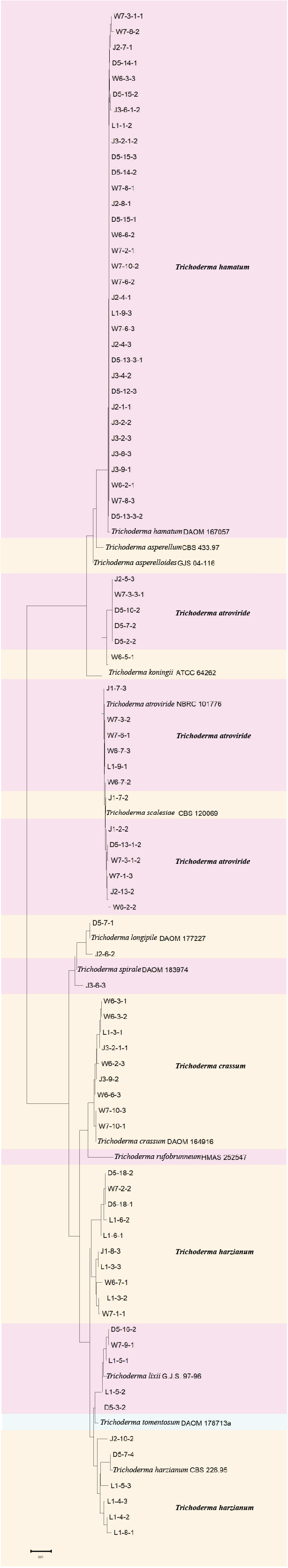

Supplement: Supplementary file 1 [file Image_1.JPEG]
